# Renoprotective effects of GHRH agonist MR409 is associated with reduced oxidative stress and ferroptosis in diabetic mice

**DOI:** 10.3389/fphar.2025.1617185

**Published:** 2025-08-25

**Authors:** Yueyang Liu, Rong Fu, Qi Tang, Yaoxia Zhang, Ruiping Cai, Limin Liu, Hui Jia, Junjia Gao, Ming-Sheng Zhou

**Affiliations:** 1 Shenyang Key Laboratory of Vascular Biology, Science and Experimental Research Center of Shenyang Medical College, Shenyang, China; 2 Department of Physiology, Shenyang Medical College, Shenyang, China; 3 Department of Cardiology, 2nd Affiliated Hospital Shenyang Medical College, Shenyang, China; 4 School of Traditional Chinese Medicine, Shenyang Medical College, Shenyang, China

**Keywords:** growth hormone-release hormone analogue, diabetic nephropathy, oxidative stress, ferroptosis, Klotho

## Abstract

MR409, a synthetic growth hormone-releasing hormone (GHRH) analogue, has demonstrated therapeutic potential in enhancing islet cell transplantation efficacy in diabetes mice and exerts beneficial effects on cardiovascular diseases. The present study investigated the renoprotective effects of MR409 on db/db and streptozotocin (STZ)-induced diabetic mice, focusing on its role in modulating oxidative stress and ferroptosis. db/db or STZ mice combined with high fat diet were used to establish the type 2 diabetic models. MR409 (15 μg/mouse/day) was subcutaneously administrated for 8 weeks. Treatment with MR409 significantly improved renal function, reduced the renal injury and fibrosis in both db/db and STZ-induced diabetic mice. MR409 increased the expression of renal GHRH receptor without affecting plasma level of the growth hormone. It attenuated oxidative stress, evidenced by decreased expressions of NADPH oxidase subunits p22phox, gp91phox, reduced dihydroethidium oxidative fluorescence intensity, and lowered renal expression of malondialdehyde and 4-hydroxynonenal in db/db mice. Meanwhile, MR409 inhibited ferroptosis, as indicated by upregulating the expressions of glutathione peroxidase 4, nuclear factor erythroid 2-related factor, ferritin heavy chain and downregulating transferrin receptor expression, alongside restoring renal glutathione level in db/db mice. Notably, MR409 activated the peroxisome proliferator-activated receptor γ and its downstream targeted gene Klotho in diabetic kidney. Collectively, the present study demonstrated that MR409 alleviates diabetic nephropathy, mitigates oxidative stress and ferroptosis, offering a novel therapeutic insight for diabetic nephropathy.

## 1 Introduction

Diabetic nephropathy (DN) is a prevalent microvascular complication of diabetes mellitus (DM) and the leading cause of end-stage renal disease (ESRD) worldwide, with an approximately 40% incidence of subjects with diabetes (Magee et al., 2017; Peters et al., 2020). Currently, available therapeutic options for DN patients are limited, which include angiotensin converting enzyme inhibitors (ACEIs), angiotensin II receptor antagonists (ARBs), and sodium-dependent glucose transporter 2 (SGLT-2) inhibitors. These agents to some extents delay but cannot halt the progression of DN (Ricciardi and Gnudi, 2021; Chen et al., 2023). A large portion of DN patients ultimately develop ESRD and rely solely on dialysis or kidney transplantation. One of main reasons for the lack of effective therapy in DN patients is our finite understanding of DN pathogenesis.

The mechanisms of DN are very complex, including hyperglycemia, hyperlipidemia, insulin resistance, oxidative stress and inflammation, among them, hyperglycemia is the primary culprit (Habib, 2013). Persistent hyperglycemia exerts a metabolic stress on vascular and renal cells to activate multiple cellular signaling pathways, which induce mitochondria dysfunction, oxidative stress and inflammation, even cell death. Increasing evidence has shown that hyperglycemia increases reactive oxygen species (ROS) production and reduces antioxidant capacity in the renal cells (Rosa et al., 2009; Yeh et al., 2016), resulting in an imbalance between renal oxidant and antioxidant. Oxidative stress promotes renal inflammation, extracellular matrix (ECM) deposition, and fibrosis by activating its downstream signaling, such as nuclear factor (NF)*κ*B, transforming growth factor (TGF)-β/Smad, and phosphoinositide 3-kinase (PI3K)/Akt signaling pathways, which can be reflected in the main pathological alternations of DN, including the thickening of renal tubules and glomerular basement membrane, mesangial expansion and renal interstitial fibrosis (Gu et al., 2021).

One of most deteriorating effects caused by oxidative stress is renal cell death, including ferroptosis. Ferroptosis is a distinct nonapoptotic type of regulated cell death caused by intracellular iron overload-dependent massive ROS production and lipid peroxides in cell membrane. Ferroptosis has been recently demonstrated to be involved in the pathogenesis of DN (Dixon et al., 2012). Several studies have shown that the initiation and development of DN are influenced by ferroptosis (Wang et al., 2020; Kim et al., 2021; Li et al., 2021). Ferrostatin-1(Fer-1), a synthetic antioxidant compound, can inhibit ferroptosis by reducing ROS levels, removing cellular unstable iron, and depleting lipid peroxides (Miotto et al., 2020).

Growth hormone-releasing hormone (GHRH) is a neuropeptide predominantly secreted by the hypothalamus. GHRH binds to GHRH receptor (GHRHR) in the pituitary gland to release growth hormone and regulate body growth and development. In addition to its expression in the pituitary cells, GHRHRs are also expressed in various non-pituitary cells, such as the heart, kidney, pancreatic islets, and retina (Kiaris et al., 2011). Notably, an increasing number of studies have shown that GHRH agonist MR-409 has beneficial effects in experimental myocardial infarction, heart failure and ischemia-induced brain injury (Gesmundo et al., 2017), and enhances the survival rate and efficacy of islet cell transplantation in streptozotocin (STZ)-induced type 1 diabetes mice (Zhang et al., 2015). In addition, MR409 has been shown to have potent antioxidant and anti-inflammatory effects. In culture vascular smooth muscle cells, MR409 inhibits NADPH oxidase-derive ROS to suppress vascular calcification (Shen et al., 2018), we have recently shown that long-term treatment with MR-409 slows down vascular calcification in db/db diabetic mice through upregulating Klotho, which inhibits vascular ROS production (Ren et al., 2023).

This study investigated the renoprotective effects of MR-409 in two models of DN: db/db mice and STZ-induced diabetic mice, with a focus on exploring its potential mechanisms related to oxidative stress and ferroptosis pathways.

## 2 Materials and methods

### 2.1 Animal protocols

We established 2 mouse models of diabetes: leptin receptor-deficient db/db diabetic mice or STZ-induced diabetic mice. All animal procedures were carried out in accordance with the National Institutes of Health (NIH) guidelines for the Care and Use of Laboratory Animals and approved by the Ethics Committee of Shenyang Medical College (SYYXY2021032301). Ten-weeks-old male db/db mice on a C57BLKS/J background were purchased from the Model Animal Research Center of Nanjing University (Nanjing, Jiangsu, China) and housed under specific pathogen-free conditions in the Laboratory Animal Center of Shenyang Medical College (Shenyang, Liaoning, China), control mice were littermate heterozygous (db/+). After a 2-week acclimatization period, db/db mice or db/m mice were divided into three groups: a control group (control, n = 10), a db/db diabetic group (db/db, n = 10) and db/db mice treated with MR409 group (db/db + MR409, n = 10). All group mice were fed a regular mouse diet. The mice on db/db + MR409 group received subcutaneous injection of MR-409 at 15 μg/mouse/day, with MR409 dissolved in a 10% of 1, 2-propanediol solution. This dose was selected based on our prior studies demonstrating its efficacy in treating diabetic vascular complications and chronic ischemic infarction without adverse effects in mice (Liu et al., 2021; Ren et al., 2023; Liu et al., 2025). The mice in control group and db/db group were subcutaneously injected with an equivalent volume of vehicle solution.

To establish STZ-induced type II diabete or DN model, 10-week-old C57BL/6J mice were fed a high-fat diet (45% kcal fat, 20% kcal protein and 35% kcal carbohydrates) for 2 weeks. Mice then received intraperitoneal injection of STZ at 50 mg/kg/day dissolved in citrate buffer for five consecutive days while maintaining the high fat diet. Control mice received equivalent volume of citrate solution injection and were fed a regular mouse diet (17% kcal fat). Fasting blood glucose levels were monitored every 3 days post-STZ injection until levels exceeded 250 mg/dL, confirming the induction of diabetes. Diabetic mice were randomized into two groups: the STZ diabetic group and STZ + MR409 group. MR409 administration followed the same protocol as described for db/db mice. Throughout the experiment, both STZ and STZ + MR409 groups remained on the high-fat diet, while the control group continued the regular mouse diet.

Body weight was measured weekly, while fasting blood glucose was assessed biweekly using an automatic blood glucose monitoring system (Roche Accu-CHEK Active, Mannheim, Germany). Urine samples were obtained by gently compressing the mouse bladder over a metal plate to stimulate urination. Urine albumin was quantified using the Bio-Rad protein assay (Beyotime Biotech, Shanghai, China), and urine levels of creatinine were determined with a creatinine assay kit (Nanjing Jiangchen Bioengineering Institute Co, Nanjing, China), respectively, in accordance with the manufacturer’s protocols. The urine albumin excretion was expressed as albumin-to-creatinine ratio.

At the end of the study, mice were subjected to an overnight fasting period and subsequently anesthetized with a combination of 100 mg/kg ketamine and 20 mg/kg xylazine. Following anesthesia, the thoracic cavity was promptly opened, and blood samples were harvested through left ventricular puncture. These blood samples were used to measure plasma levels of total cholesterol (TC) and total triglycine (TG). The kidneys were excised, weighted, and the renal index (mg/g) was calculated as the ratio of kidney weight (mg) to body weight (g), Then renal tissue samples were snap frozen with liquid nitrogen and stored in −80 °C freezer.

### 2.2 Biochemical analysis

Serum levels of total TC and TG were quantified using the GPO-PAP enzymatic method and COD-PAP method, respectively. The serum GH concentration was measured using a mouse GH ELISA Kit (CUSABIO, Wuhan, China), according to the manufacturer’s instructions. In brief, samples were incubated in microtitration wells pre-coated with an anti-mouse GH antibody. A standard curve for the assay was generated using reference samples supplied by the manufacturer. The optical density measurements were obtained using a microplate reader.

### 2.3 Renal histology

Kidney tissues were fixed in 10% neutral buffered formalin, embedded in paraffin, and sectioned at a thickness of 3 μm. Following deparaffinization and hydration, the sections were stained with hematoxylin-eosin (H&E) for general histological evaluation, periodic acid-Schiff (PAS) to assess glomerulosclerosis and glycogen deposition, and Masson’s trichrome and Sirius red stains to evaluate collagen deposition and interstitial fibrosis, respectively. Quantitative assessment of the fibrotic areas was performed by measuring the positive stained areas in Masson’s trichrome and Sirius red stained sections using the ImageJ software (NIH, United States). Similarly, glycogen deposition was quantified by analyzing the positively stained areas in PAS-stained sections with the same software. All procedures were conducted in accordance with established protocols and standardized methodologies.

### 2.4 Determination of ROS with dihydroethidium (DHE) fluorescence oxidative staining

Renal ROS was determined by dihydroethidium (DHE) fluorescence oxidative staining, as previously described with modifications (Ren et al., 2023). Briefly, paraffin-embedded tissue sections were deparaffinized and rehydrated using standard protocols. The sections were then incubated with a DHE fluorescent probe (Sigma Aldrich; 37,291), prepared by dissolving in dimethyl sulfoxide (DMSO), at a concentration of 10 µM. Incubation was carried out at 37 °C in a light-protected environment for 30 min to allow for the oxidation of DHE by intracellular ROS. After incubation, the samples were rinsed with PBS, and stained sections were stored at 4 °C, with fluorescence quantification was completed within 30 min. Fluorescence signals were visualized and captured using an inverted fluorescence microscope equipped with appropriate excitation (488 nm) and emission (610 nm) filters. Image analysis was performed using ImageJ software to quantify fluorescence intensity per unit square area, which correlates with ROS levels (Xia et al., 2024).

### 2.5 Determination of glutathione (GSH) levels

The GSH content in the kidney tissues was examined using the Micro-reduced GSH Assay Kit (BC1175, Solarbio). As the manufacturer’s construction, and the content of each sample was identified with a microplate reader at the wavelength at 412 nm. The GSH levels were normalized by protein amount in kidney homogenates.

### 2.6 Immunofluorescence and immunohistochemistry staining

To evaluate GHRHR expression in kidney tissues, immunofluorescence staining was performed. Paraffin-embedded tissue sections were deparaffinized and rehydrated using standard protocols. Antigen retrieval was conducted by incubating the sections in sodium citrate buffer (pH 6.0) at 95 °C for 20 min. Non-specific binding sites were blocked with 5% bovine serum albumin (BSA) in PBS for 1 h at room temperature. The sections were then incubated overnight at 4 °C with a primary antibody against GHRHR (Abcam, Cambridge, MA, United States; 1:200 dilution). After washing with PBS, the sections were incubated with an Alexa Fluor 488-conjugated secondary antibody (Invitrogen, United States) for 1 h at room temperature in the dark. Nuclei were counterstained with 4′,6-diamidino-2-phenylindole (DAPI) (Sigma-Aldrich, United States). Fluorescence signals were visualized and analyzed using a confocal laser scanning microscope (Leica or Zeiss, Germany).

To assess ferroptosis markers, immunohistochemical staining for malondialdehyde (MDA) and 4-hydroxynonenal (4-HNE) was performed. Deparaffinized and rehydrated tissue sections underwent antigen retrieval using sodium citrate buffer (pH 6.0) at 95 °C for 20 min. Endogenous peroxidase activity was quenched by incubating the sections with 3% hydrogen peroxide (H_2_O_2_) for 10 min at room temperature. The sections were then incubated overnight at 4 °C with primary antibodies against MDA (ab243066, Abcam, Cambridge, MA, United States; 1:200 dilution) and 4-HNE (ab48506, Abcam, Cambridge, MA, United States; 1:200 dilution), followed by incubation with a horseradish peroxidase (HRP)-conjugated secondary antibody (Vector Laboratories, United States) for 10 min at room temperature in the dark. Immunoreactivity was visualized using 3,3′-diaminobenzidine (DAB), followed by counterstaining with hematoxylin. Stained sections were analyzed, and images were captured using a light microscope (Nikon or Olympus, Japan).

### 2.7 Western blotting

Total protein was extracted from kidney tissues using RIPA lysis buffer supplemented with protease inhibitors. Protein concentrations were determined using a bicinchoninic acid (BCA) assay kit (Thermo Fisher Scientific, United States). Equal amounts of protein (20 μg) were separated by appropriate concentration of SDS-PAGE gel and subsequently transferred onto polyvinylidene difluoride (PVDF) membranes via electroblotting. The membranes were blocked with 5% non-fat milk in Tris-buffered saline containing 0.1% Tween-20 (TBST) for 1 h at room temperature to prevent nonspecific binding. Following blocking, the membranes were incubated overnight at 4 °C with the following primary antibodies: anti-p22phox (sc-271262), anti-Fibronectin (sc-271098), and anti-TGFβ1 (sc-146) from Santa Cruz Biotechnology; anti-phospho-endothelial nitric oxide synthase (eNOS at Ser1177, AF3247) from Affinity Biosciences; anti-peroxisome proliferator-activated receptors γ (PPARγ, #2435) from Cell Signaling Technology; anti-gp91phox (ab80508), anti-GHRHR (ab76263) and anti-Klotho (ab181373) from Abcam; and anti-β-actin (AC004) from AB clonal. After incubation, the membranes were washed three times with TBST and incubated with HRP-conjugated secondary antibodies for 1 h at room temperature. Protein bands were visualized using an enhanced chemiluminescence (ECL) detection system (Bio-Rad, United States) and quantified using ImageJ software.

### 2.8 RNA extraction, library construction and sequencing

Total RNA was isolated from mouse kidney tissues using the Trizol reagent (Invitrogen, Carlsbad, CA, United States) following the manufacturer’s protocol. RNA integrity and quality were assessed using an Agilent 2,100 Bioanalyzer (Agilent Technologies, Palo Alto, CA, United States) and verified by RNase-free agarose gel electrophoresis. Eukaryotic mRNA was subsequently enriched from the total RNA using Oligo (dT) magnetic beads, while prokaryotic rRNA was depleted using the Ribo-Zero™ Magnetic Kit (Epicentre, Madison, WI, United States). The enriched mRNA was fragmented into short segments using a fragmentation buffer and reverse-transcribed into first-strand cDNA using random primers. Second-strand cDNA synthesis was performed using DNA polymerase I, RNase H, dNTPs, and reaction buffer. The resulting cDNA fragments were purified using the QiaQuick PCR extraction kit (Qiagen, Venlo, Netherlands), followed by end repair, poly(A) tailing, and ligation to Illumina sequencing adapters. The adapter-ligated cDNA libraries were size-selected by agarose gel electrophoresis, amplified by PCR, and subjected to high-throughput sequencing on the Illumina HiSeq2500 platform (Illumina, San Diego, CA, United States) at Gene *Denovo* Biotechnology Co. (Guangzhou, China). Sequencing data were processed to quantify gene abundance, followed by pathway enrichment analysis and Gene Ontology (GO) functional enrichment analysis of differentially expressed genes. These analyses were conducted using established bioinformatics pipelines and software tools.

### 2.9 Statistical analysis

Statistics analysis was carried out with SPSS 24.0 and GraphPad Prism 8. The results from at least three independent experiments were expressed as mean ± SEM. Comparisons between different groups were performed using one-way ANOVA followed by Fisher’s LSD for multiple comparisons between groups. A p value <0.05 was considered statistically significant.

## 3 Results

### 3.1 MR409 improves renal function and structural injury in db/db and STZ-induced diabetic mice

**FIGURE 1:**
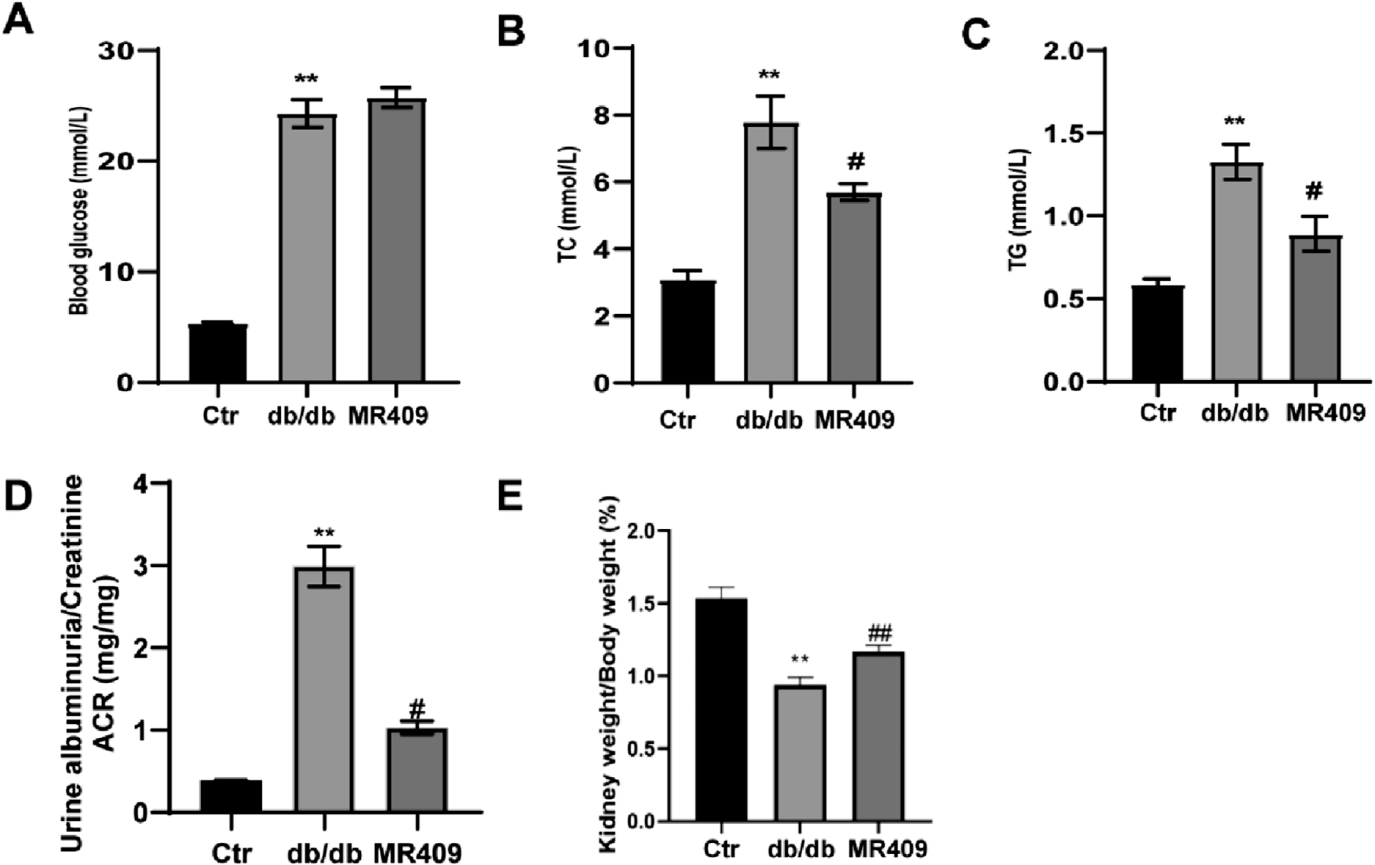
Effects of MR409 on fasting blood glucose **(A)**, serum level of total cholesterol [TC, **(B)**] and total triglycine [TG, **(C)**] the ratio of albuminuria to creatinine [ACR, **(D)**] renal index **(E)** in db/db mice. Data are expressed as mean ± SEM. ^**^ *P* < 0.01 vs control (Ctr) group; ^##^ *P* < 0.01, ^#^ *P* < 0.05 vs db/db group. n = 6.

To evaluate the effects of MR409 on renal function, renal parameters and blood glucose level were measured in db/db diabetic mice. Blood glucose level was significantly elevated in db/db mice; however, treatment with MR409 did not alter blood glucose level (Figure 1A). Compared to control mice, db/db mice exhibited significantly higher serum levels of total TC, TG, and the urine albumin-to-creatinine ratio. These metabolic and renal abnormalities were mitigated following MR409 treatment (Figures 1B–D). The kidney index, calculated by the ratio of kidney weight to body weight, was markedly reduced in db/db mice compared to control mice, but was normalized by MR409 administration (Figure 1E).

**FIGURE 2:**
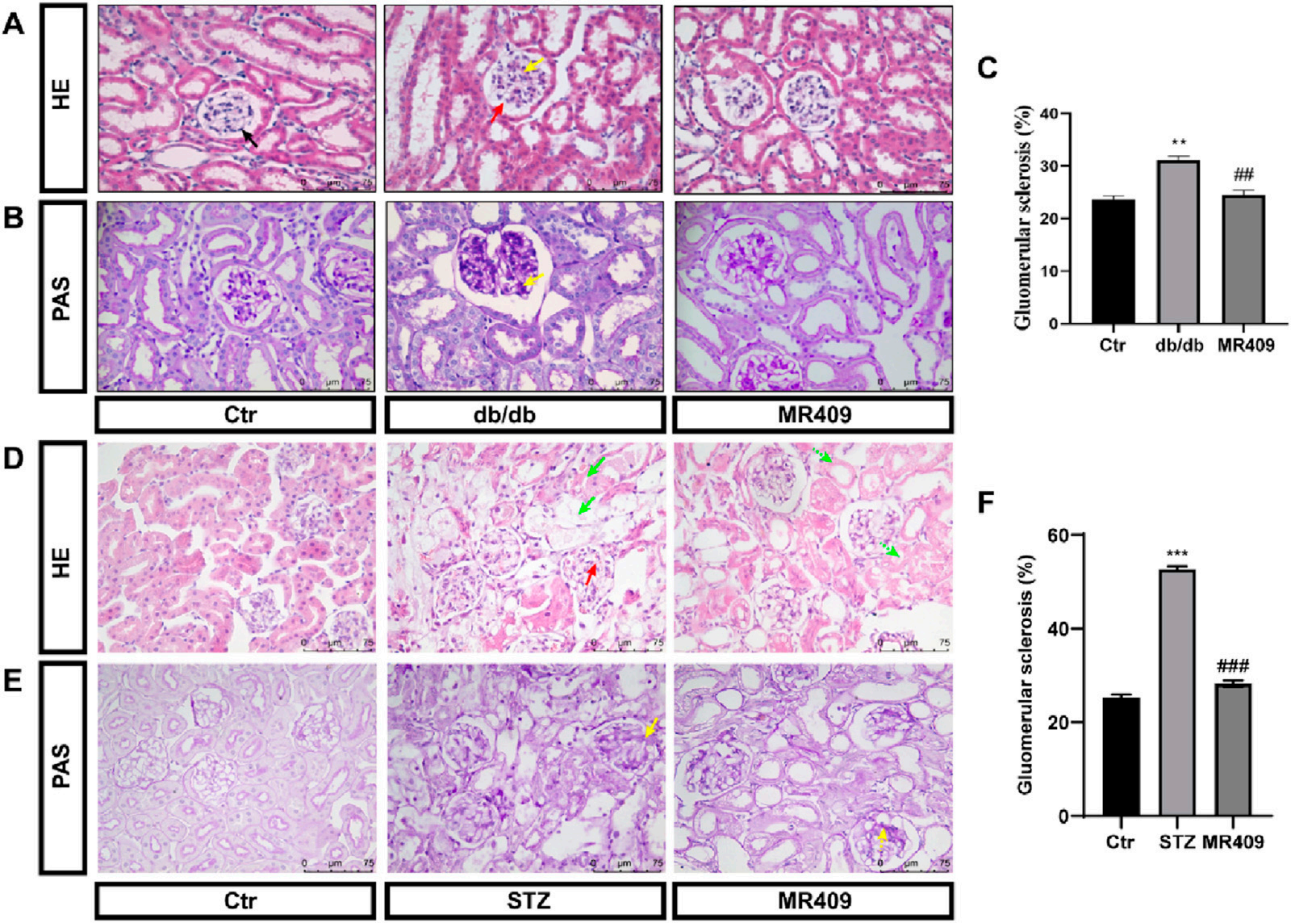
MR409 attenuates renal structural injury in db/db and STZ-induced diabetic mice. Representative images of renal sections with HE staining **(A)** and PAS staining **(B)** in db/db mice, Semiquantitative analysis of glomerular sclerosis from PAS staining **(C)**. Representative images of renal sections with HE staining **(D)** and PAS staining **(E)** in STZ-induced diabetic mice, **(F)** Semiquantitative analysis of glomerular sclerosis in STZ mice. Black arrow: normal glomerulus; Red arrow: Glomerular hypertrophy; Yellow arrow: renal mesangial matrix proliferation and expansion; Green arrow: vacuolar degeneration of renal tubular epithelial cells, disappearance of nuclei, and partial cell disintegration. The dashed arrows of corresponding colors represent the improvement of renal pathological damage in MR409-treated mice. Data are expressed as mean ± SEM. ^***^ *P* < 0.001, ^**^ *P* < 0.01 vs control (Ctr) group; ^###^ *P* < 0.001, ^##^ *P* < 0.01 vs db/db or STZ group. n = 6. Scale bar = 75 μm in panels **(A–E)**.

Furthermore, renal histopathology was evaluated using H&E and PAS staining in both db/db and STZ-induced diabetic mice. H&E staining revealed a well-organized architecture of glomeruli, renal tubules, and mesangium with intact basement membranes in normal control mice. In contrast, db/db and STZ diabetic mice displayed glomerular dilatation, increased mesangial matrix deposition, cystic wall cell proliferation, and partial renal tubular atrophy. These pathological changes were ameliorated by MR409 treatment (Figures 2A,D). PAS staining indicated a significant increase in the percentage of glomerular sclerosis in db/db and STZ mice, which was reduced by MR409 (Figures 2B,C,E,F).

**FIGURE 3:**
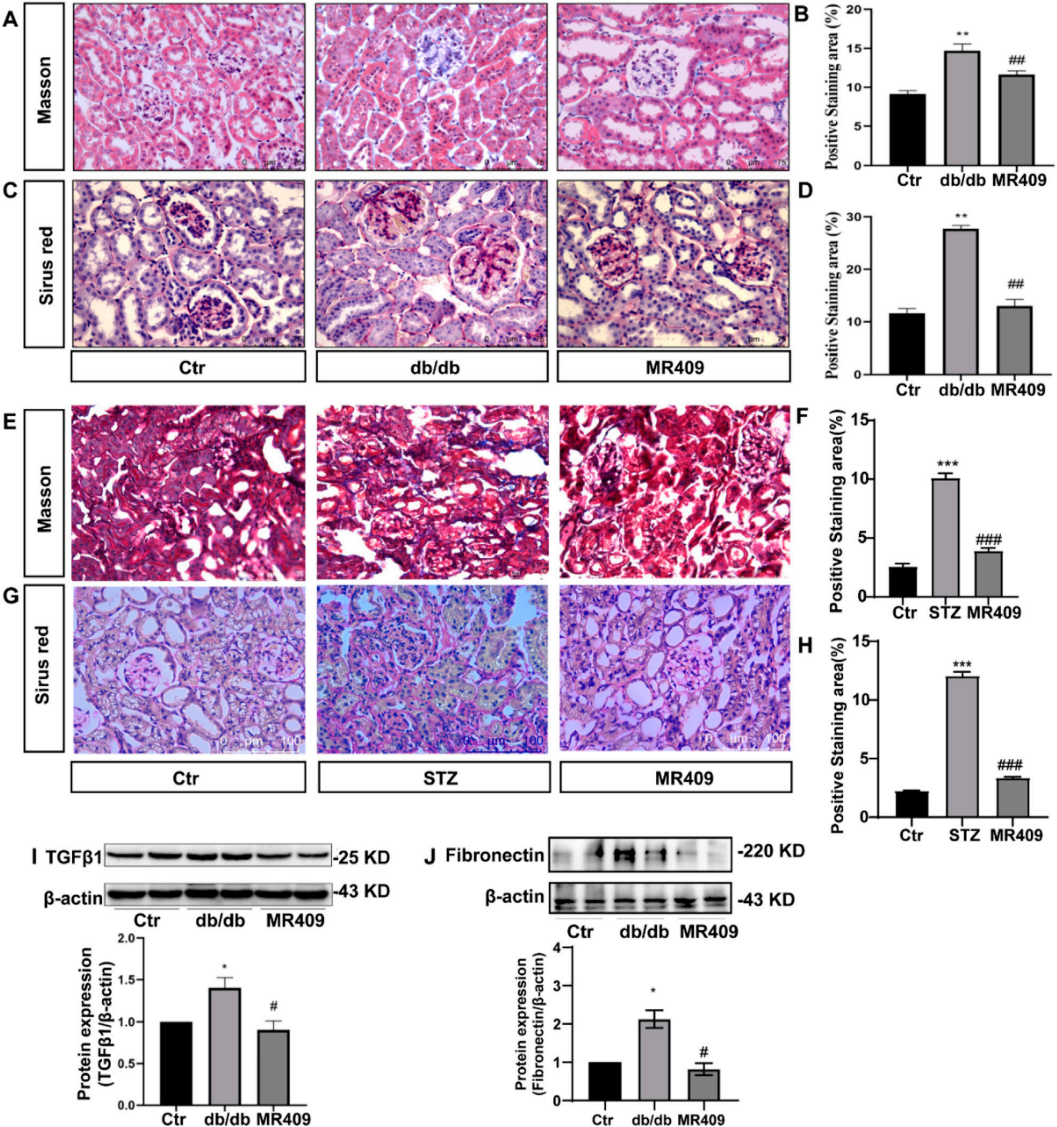
MR409 attenuates the renal fibrosis in db/db and STZ-induced diabetic mice. Representative images of renal sections from db/db mice by Masson staining **(A)** and Sirus red staining **(C)**, and correspondence semiquantitative analysis of positive staining area **(B,D)**. Representative images of renal sections from STZ mice by Masson staining and Sirus red staining **(E,G)**, and correspondence semiquantitative analysis of positive staining area **(F,H)**. Protein expressions of TGFβ1 and Fibronectin in the kidney of db/db mice **(I,J)**. Data are expression as mean ± SEM. ***P < 0.001, **P < 0.01, *P < 0.05 vs control (Ctr) group; ^###^ *P* < 0.001, ^##^ *P* < 0.01, ^#^ *P* < 0.05 vs db/db group or STZ group. n = 6. Scale bar = 75 μm in panels **(A,C,G)**, Scale bar = 100 μm in panels **(E)**.

To investigate the effects of MR409 on renal fibrosis in both db/db and STZ-induced diabetic mice. Masson’s trichrome staining and Sirius red staining were used to assess the extent of collagen deposition. Both methods revealed a significant increase in the positively stained areas for collagen fibers in db/db and STZ diabetic mice (Figures 3A–H). However, these changes were attenuated following treatment with MR409 (Figures 3A–H). Given the established role of the TGFβ1 signaling pathway in the pathogenesis of renal fibrosis (Liu et al., 2019), the expression levels of TGFβ1 and its downstream effector, Fibronectin, were determined in the kidneys of db/db mice by Western blot. The results indicated a marked upregulation of renal TGFβ1 and Fibronectin, which was significantly downregulated by MR409 treatment (Figures 3I,J). Collectively, these findings demonstrate that MR409 improves renal function and structural damage in diabetic mice.

### 3.2 MR409 upregulates renal GHRHR expression without altering plasma GH level in db/db diabetic mice

**FIGURE 4:**
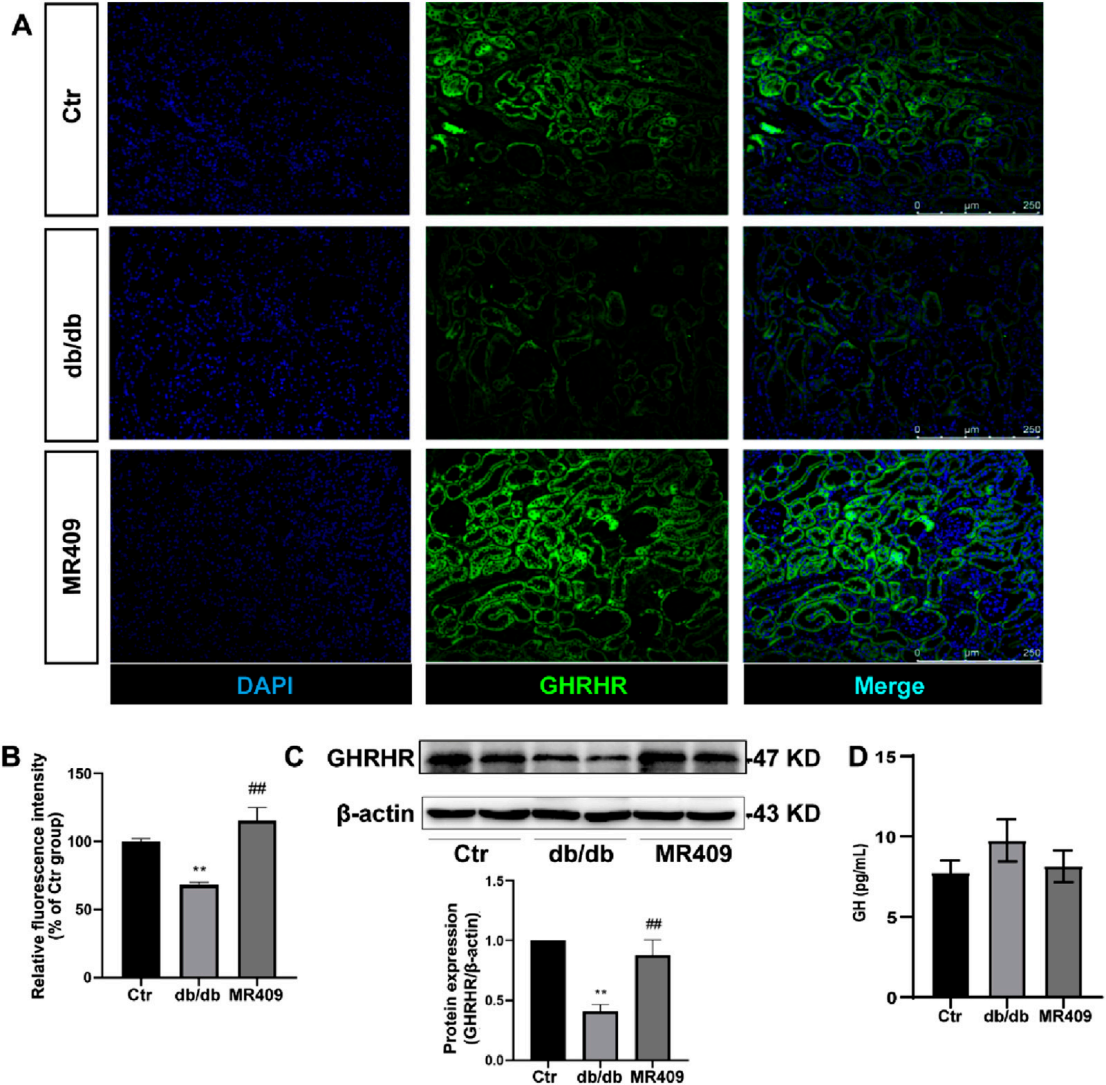
MR409 upregulates GHRHR expression in the kidney of db/db mice. **(A)** Representative images of GHRHR in the kidney of db/db mice stained by immunofluorescence. **(B)** Semi-quantitative analysis of the relative fluorescence intensity. **(C)** Protein expression of GHRHR. **(D)** Serum level of GH. Data are expression as mean ± SEM. ^**^ *P* < 0.01 vs control (Ctr) group; ^##^ *P* < 0.01 vs db/db group. n = 6. Scale bar = 250 μm.

As MR409 is a potent GHRHR agonist analog, its effects on the expression of GHRHR and GH levels were evaluated. Immunostaining revealed a significant reduction in the relative fluorescence intensity of GHRHR in the kidneys of db/db mice, which was partially restored following MR409 treatment (Figures 4A,B). Consistent with this finding, Western blot analysis demonstrated a marked downregulation of renal GHRHR expression in db/db mice, which was upregulated by MR409 administration (Figure 4C). Notably, GH levels remained unchanged across control, db/db mice, and MR409-treated db/db mice (Figure 4D). These findings indicate that MR409 enhances renal GHRHR expression in db/db mice without influencing circulating GH levels.

### 3.3 MR409 attenuates renal oxidative stress in db/db mice

**FIGURE 5:**
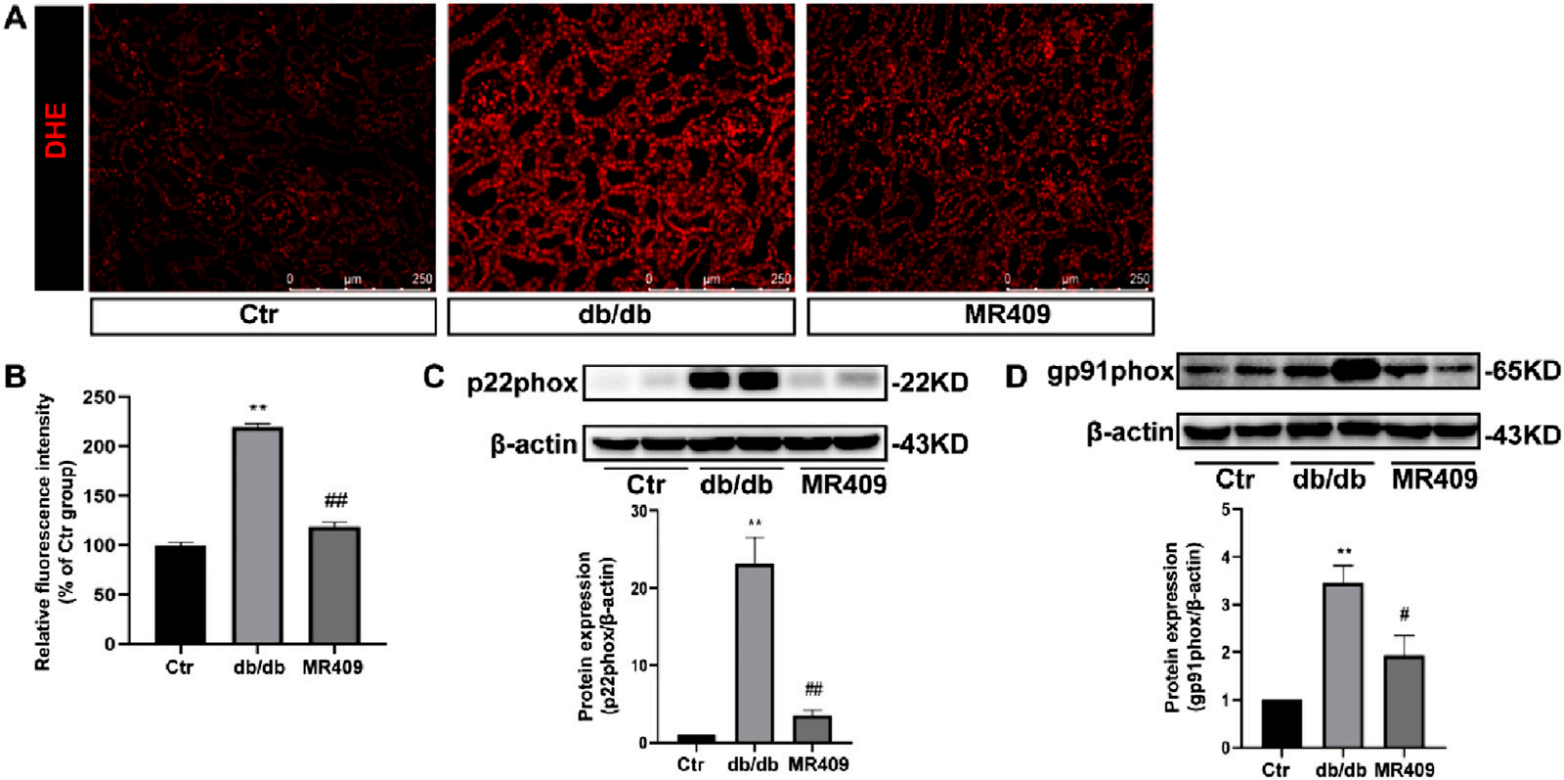
MR409 attenuates oxidative stress in the kidney of db/db mice. **(A)** Representative images of ROS oxidative fluorescence intensity in the kidney of db/db mice stained by DHE. **(B)** Quantitative analysis of the relative fluorescence intensity. **(C,D)** Protein expressions of p22phox and gp91phox. Data are expression as mean ± SEM. ^**^ *P* < 0.01 vs control (ctr) group; ^##^ *P* < 0.01, ^#^ *P* < 0.05 vs db/db group. n = 6. Scale bar = 250 μm.

Renal ROS generation was assessed using DHE staining, this staining demonstrated significantly increased fluorescence intensity in the kidney of db/db mice compared to control, and an effect attenuated by MR409 treatment (Figures 5A,B). Given NADPH oxidase is a main source of renal ROS production, we evaluated the expressions of its membrane-bound subunits gp91phox and p22phox. Western blot analysis showed significant upregulation of both subunits of gp91phox and p22phox in the kidneys of db/db mice, which was attenuated by MR409 treatment (Figures 5C,D). These data demonstrate that MR409 attenuates renal oxidative stress in db/db mice.

### 3.4 MR409 attenuates ferroptosis in the kidneys of db/db mice

**FIGURE 6:**
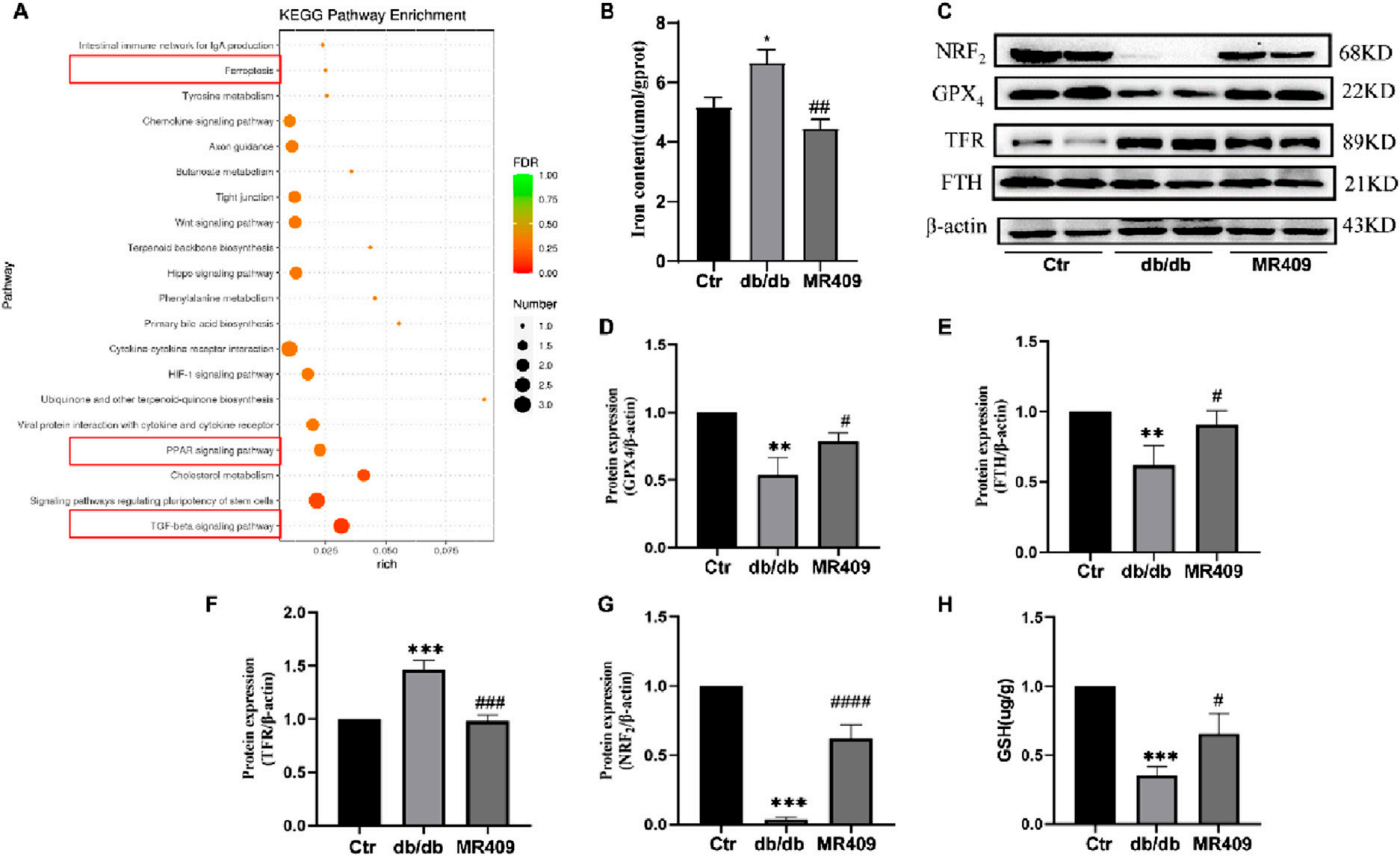
MR409 reduces ferroptosis in the kidney of db/db mice. **(A)** KEGG enrichment analysis showed the top 20 enriched pathways that affected by MR409 in db/db mice. **(B)** Renal iron content. **(C)** Representative images of Westen blot bands and semiquantitative analysis of the expressions of ferroptosis related proteins GPX4 **(D)**, FTH **(E)**, TFR **(F)**, and NRF2 **(G)**. **(H)** Renal GSH content. Data are expression as mean ± SEM. ^***^ *P* < 0.001, ^**^ *P* < 0.01, ^*^ *P* < 0.05 vs control (ctr) group; ^###^ *P* < 0.001, ^##^ *P* < 0.01, ^#^ *P* < 0.05 vs db/db group. n = 6.

To elucidate the mechanism underlying MR409-mediated renoprotection in db/db mice, we conducted a broad-spectrum gene screening in kidney tissues using RNA-sequencing analysis. KEGG pathway enrichment analysis revealed the top 20 enriched pathways, with the ferroptosis-related pathway being significantly enriched in MR409-treated db/db mice compared to untreated db/db mice (Figure 6A).

**FIGURE 7:**
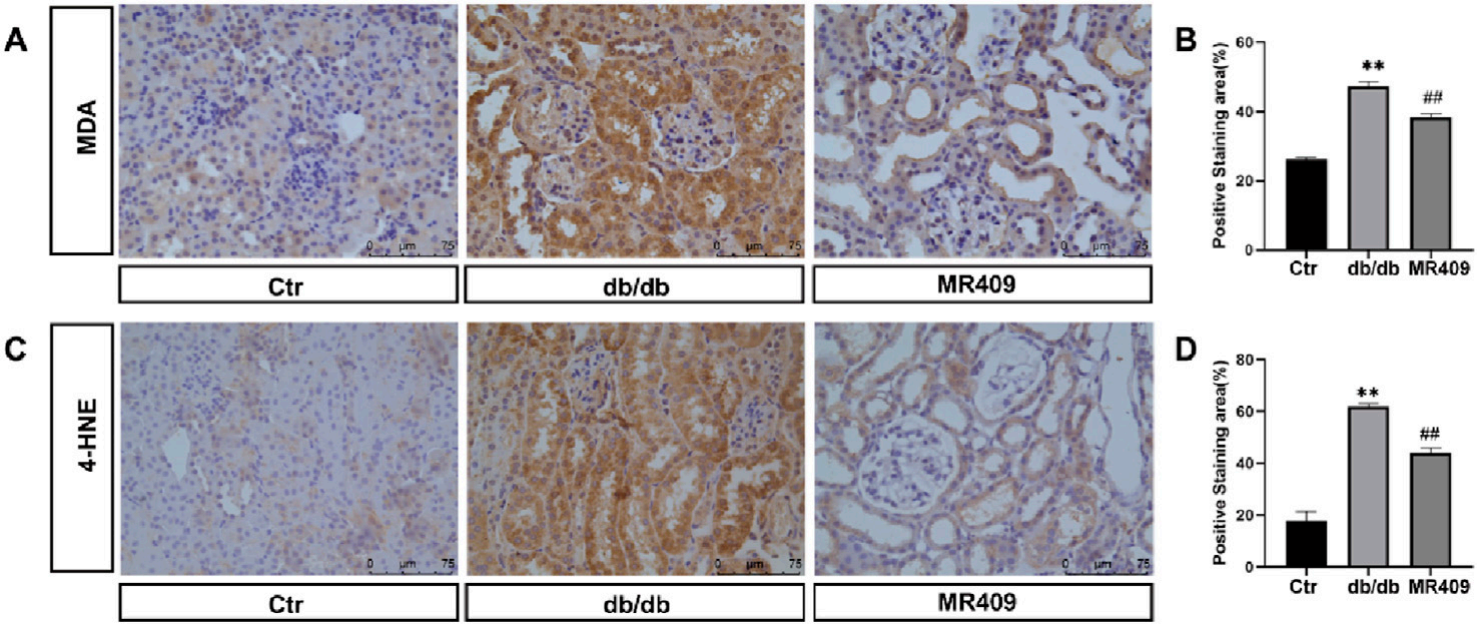
MR409 reduces ferroptosis in the kidney of db/db mice. Representative images showing the effects of MR409 on the level of MDA **(A)** and 4-HNE **(C)** in the kidney of db/db mice by immunohistochemistry. **(B)** Histogram showing the relative staining area of MDA **(B)** and 4-HNE **(D)**. Data are expression as mean ± SEM. ^**^ *P* < 0.01 vs control (ctr) group; ^##^ *P* < 0.01, vs db/db group. n = 6. Scale bar = 75 μm in panels **(A,C)**.

Ferroptosis, a distinct form of regulated cell death characterized by iron overload and accumulation of lipid peroxides, has been implicated in the pathogenesis of DN (Li et al., 2024). We measured the iron content and observed a significant increase in the kidney of db/db mice, which was downregulated by MR409 treatment (Figure 6B). Western blot analysis demonstrated downregulation of ferroptosis suppressors glutathione peroxidase 4 (GPX4) and ferritin heavy chain (FTH) (Figs. C–E), alongside upregulation of the ferroptosis promoter transferrin receptor (TFR) in the kidney of db/db mice (Figs C &F). These alterations were partially reversed by MR409 treatment (Figures 6C–F). Given the established role of nuclear factor erythroid 2-related factor (NRF2) signaling pathway in regulating ferroptosis (Dodson et al., 2019). We assessed NRF2 levels and observed a significant reduction in NRF2 levels in DN mice, which was rescued by MR409 treatment (Figure 6G). Furthermore, MR409 restored depleted renal GSH content in db/db mice (Figure 6H). Critically, MDA and 4-HNE represent two important biomarkers of lipid peroxides and ferroptosis. The expressions of MDA and 4-HNE were significantly increased in the kidney of db/db mice, and these changes were significantly reduced by MR409 treatment (Figures 7A–D). Collectively, these findings demonstrate that MR409 attenuates renal injury and oxidative stress in db/db mice, likely through the inhibition of ferroptosis.

### 3.5 MR409 activates the Klotho signaling pathway

**FIGURE 8:**
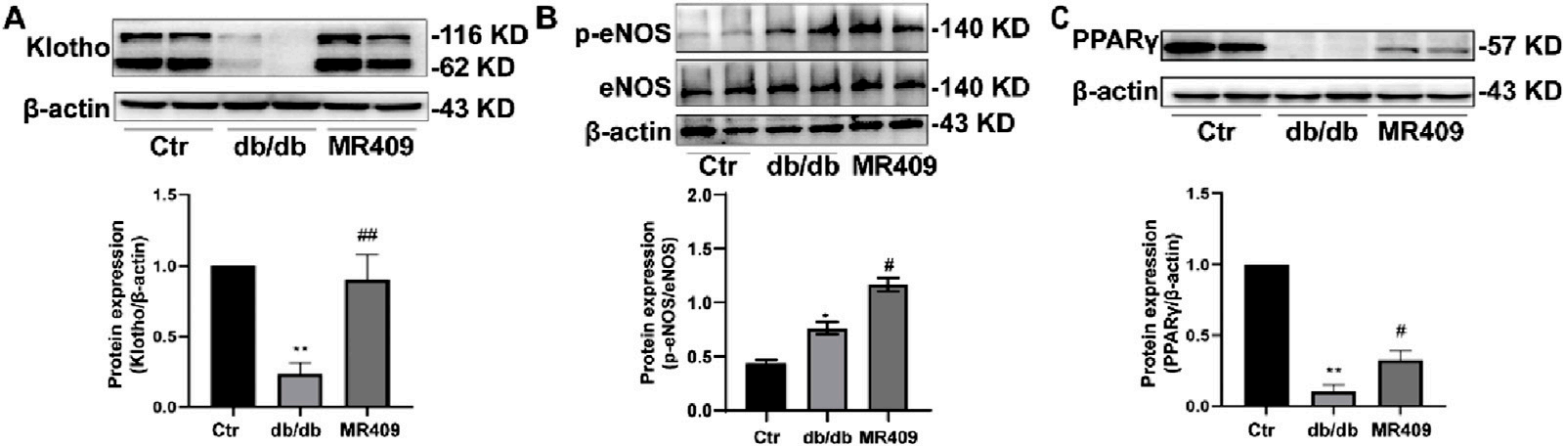
MR409 prevented diabetes-induced decrease in the protein expression of Klotho **(A)**, p-eNOS **(B)** and PPARγ **(C)** in the kidney of db/db mice. Data are expression as mean ± SEM. ^**^ *P* < 0.01, ^*^ *P* < 0.05 vs control (ctr) group; ^##^ *P* < 0.01, ^#^ *P* < 0.05 vs db/db group. n = 6.

Klotho, an anti-aging protein downregulated in DN, exerts protective effects in DN pathogenesis (Wu et al., 2022). Recent evidence indicates that Klotho may mitigate DN by inhibiting oxidative stress and ferroptosis (Qian et al., 2018). Consistent with these reports, we observed a significant reduction in Klotho expression in the kidneys of db/db mice, which was upregulated by MR409 treatment (Figure 8A). Furthermore, p-eNOS was elevated in the kidneys of db/db mice and further enhanced by MR-409 (Figure 8B). Given that Klotho is a transcriptional target of PPARγ, a ligand-activated nuclear transcription factor (Zhang et al., 2008). We assessed PPARγ expression and found that MR409 upregulated the expression of PPARγ in the kidneys of db/db mice (Figure 8C). These results indicates that PPARγ/Klotho axis, potentially contributing to MR409-mediated inhibition of oxidative stress and ferroptosis.

## 4 Discussion

This study demonstrated that prolonged administration of MR409 significantly improved renal function and mitigated renal structural damage and fibrosis in two diabetic mouse models, specifically db/db mice and STZ-induced diabetic mice. MR409 effectively reduced plasma lipid levels without significantly altering blood glucose concentration. The renoprotective effects of MR409 were associated with reduced renal ROS production, improved antioxidant capacity, and modulation of renal iron homeostasis, thereby inhibiting ferroptosis. Furthermore, MR409 prevented the diabetes-mediated downregulation of the anti-aging protein Klotho and PPARγ expression. Notably, MR409 upregulated the expression of renal GHRHRs without affecting systemic growth hormone level. These findings indicate that MR409 exerts renoprotective effects through selective activation of its localized renal receptor, highlighting its potential as a novel therapeutic candidate for DN.

DN represents one of the most serious complications of DM, characterized by progressive proteinuria, a decline in glomerular filtration rate, glomerulosclerosis, and tubulointerstitial fibrosis (Jha et al., 2024). Although renin-angiotensin system and SGLT2 inhibitors have transformed the treatment landscape for DN, a significant proportion of patients continue to experience disease progression (Perkovic et al., 2019; Heerspink et al., 2020), highlighting the urgent need for novel therapeutic strategies.

GHRH, traditionally known for its role in stimulating GH secretion from the anterior pituitary, has garnered increasing attention for its extra-pituitary effects in various tissues (Schally et al., 2019). These peripheral actions are mediated through GHRHRs and its associated signaling pathways, which may play a role in glucose homeostasis, insulin sensitivity, and diabetic complications (Fridlyand et al., 2016). Previous studies have demonstrated the therapeutic potential of the GHRHR agonist MR409 in experimental models of diabetes (Zhang et al., 2015; Thounaojam et al., 2017; Louzada et al., 2023; Zhang et al., 2024). For instance, pretreatment of islet stem cells with MR409 significantly enhances cell survival and transplantation efficacy, leading to reduced blood glucose levels and improved pancreatic function in type 1 diabetic mice (Zhang et al., 2015). Additionally, MR409 has shown promise in alleviating diabetic retinopathy (Thounaojam et al., 2017).

In our study, we identified the expression of GHRHRs in renal tissue and observed a significant downregulation of these receptors in DN. Furthermore, treatment with MR409 conferred protection against renal injury and dysfunction in both db/db and STZ-induced diabetic mice. MR409 upregulated the expression of renal GHRHRs without significantly altering plasma GH levels. These findings suggest a potential association between GHRHR signaling and renal health in the context of diabetes, positioning MR409 as a promising multifaceted therapeutic agent for diabetes-related complications, including DN.

Oxidative stress is widely acknowledged as a pivotal mediator in the pathogenesis and progression of DN (Babizhayev et al., 2015). Both pharmacological and lifestyle interventions targeting oxidative stress have demonstrated efficacy in mitigating renal damage and slowing disease progression (Zimnol et al., 2020). Previous studies have shown that GHRH and its analogues can upregulate the expression of key antioxidant enzymes and suppress NADPH oxidase-derived ROS production in vascular cells (Shen et al., 2018). In the current study, MR409 significantly reduced renal expression of NADPH oxidase subunits gp91phox and p22phox, decreased ROS production, and enhanced the expression of antioxidant enzymes, including GPX4 and NRF2. The restoration of the balance between ROS production and antioxidant defenses likely contributes, at least in part, to the renoprotective effects of MR409.

Emerging evidence highlights the central role of ferroptosis in the development and progression of DN. Ferroptosis is characterized by reduced antioxidant capacity, accumulation of redox-active iron, and elevated levels of lipid peroxidation products (Dixon et al., 2012). The downregulation of critical antioxidant enzymes such as GPX4 and NRF2, coupled with GSH depletion, are key drivers of ferroptotic cell death (Xie et al., 2016). In the context of diabetes, hyperglycemia and hyperlipidemia exacerbate renal oxidative stress and impair the antioxidant functions of GPX4 and NRF2, leading to an oxidative-redox imbalance and abnormal iron accumulation (Jiang et al., 2018). Excess redox-active iron amplifies ROS generation via the Futon reaction, further promoting lipid oxidation and the formation of lipid peroxidation products (Cao and Dixon, 2016). This cascade ultimately triggers ferroptotic cell death, contributing to the progression of DN.

GHRH is well-established for its role in promoting cell proliferation and inhibiting cell death (Siriwardana et al., 2006). However, direct evidence linking GHRH to ferroptosis remains limited. Through RNA sequencing (RNA-seq) analysis, we identified ferroptosis as one of the top 20 signaling pathways modulated by MR409 treatment. Diabetic db/db mice exhibited characteristic features of ferroptosis, including elevated levels of ROS and FTH expression, accumulation of iron-induced oxidative lipid damage products, such as MDA and 4-HNE, and reduced protein expression of key ferroptosis regulators, including GPX4, NRF2. Notably, MR409 treatment effectively reversed these ferroptosis-related alterations in the kidneys of diabetic mice. These findings suggest that MR409 mitigates renal ferroptosis concomitant with restoring the balance between oxidative stress and antioxidant defense, which may contribute to its protective effects against diabetic kidney injury.

This study further demonstrates that MR409 enhances the expression of renal Klotho, PPARγ and eNOS phosphorylation. Klotho is a multifunctional anti-aging protein with potent antioxidant properties (Olejnik et al., 2023), known to regulate iron homeostasis by modulating the expression of iron transporters and ferritin while inhibiting lipid peroxidation (Ji et al., 2024). Klotho supplementation has been shown to alleviate renal cell ferroptosis by reducing iron overload and lipid peroxidation (Tian et al., 2025). Notably, Klotho expression is downregulated in both patients and mouse models of DN. Emerging evidence indicates significant crosstalk between the PPARγ/Klotho axis and ferroptosis/ROS pathways: Klotho directly inhibits ferroptosis by modulating iron metabolism and suppressing lipid peroxidation (Tian et al., 2025), while PPARγ activation upregulates Klotho expression and attenuates NOX4-derived ROS (Bijli et al., 2015). Furthermore, we demonstrate that MR409 enhances eNOS phosphorylation at Ser1177, a key activation site predicted to increase eNOS activity and improve glomerular endothelial function. Critically, this MR409-induced eNOS activation coincides with reduced renal ROS level, contributing to its overall renoprotective effects. Our results revealed that both Klotho and PPARγ levels were significantly reduced in the kidneys of db/db mice but were restored following MR409 treatment. These findings suggest that MR409 may mitigate oxidative stress and ferroptosis, potentially via upregulating the PPARγ/Klotho pathway.

It has been shown that PPARγ signaling critically regulates macrophage polarization, which is key to fibrosis (Abdalla et al., 2020; Jiao et al., 2021a; Jiao et al., 2021b; An et al., 2023). PPARγ activation typically suppresses pro-fibrotic M2 polarization by antagonizing drivers like signal transducer and activator of transcription 6 (STAT6), potentially disrupting macrophage-fibroblast crosstalk to reduce ECM deposition. PPARγ also intersects with other fibrosis pathways: 1) It upregulates myeloid phosphatase and tensin homolog (PTEN), counteracting PI3K/Akt signaling, where PTEN loss exacerbates fibrosis (An et al., 2022); 2) PPARγ agonists inhibit pro-inflammatory cyclic GMP synthase-stimulator of interferon genes (cGAS-STING) (Ma et al., 2022; Jiao et al., 2025), reducing macrophage/myofibroblast activation. Thus, these studies suggest that MR409-mediated PPARγ-Klotho activation may orchestrate renoprotection by modulating macrophage polarization, enhancing PTEN, and suppressing cGAS-STING. However, this possibility requires further experimental confirmation.

Limitation: Several limitations warrant acknowledgment. First, although we observed concurrent attenuation of renal injury, oxidative stress, and ferroptosis with MR409 treatment, our *in vivo* approach cannot establish direct causality between these effects. The complex interplay of pathways, e.g., PPARγ-Klotho activation, reduced ROS, and suppressed ferroptosis, likely represents interconnected mechanisms rather than linear causality. Second, this study exclusively used male mice to minimize variability from the female estrous cycle; however, this design limits the generalizability of our findings to females and precludes analysis of sex-specific responses to MR409 in DN. Third, our ROS detection using DHE staining on paraffin sections, despite rigorous optimization (fresh reagent, controlled incubation, rapid quantification), may retain higher background signal compared to frozen sections due to inherent susceptibility to non-specific oxidation. While NADPH oxidase data provide complementary evidence of oxidative stress reduction, this methodological constraint should be noted. Finally, the absence of *in vitro* models restricts mechanistic granularity. Though physiologically relevant *in vivo* models are essential for evaluating integrated therapeutic effects, future studies employing renal cell types could delineate cell-autonomous actions of MR409 on identified signaling pathways. These limitations highlight avenues for further investigation.

In summary, this study highlights the renoprotective effects of MR409, a GHRHR agonist, in DN. MR409 significantly improved renal function, alleviated structural damage, and reduced fibrosis in diabetic mouse models. These results position MR409 as a promising therapeutic candidate for DN, potentially acting through multiple mechanisms, including oxidative stress reduction, ferroptosis inhibition, and restoration of the PPARγ/Klotho pathway. However, further research is needed to fully elucidate the underlying molecular mechanisms and validate these findings in clinical settings.

## Data Availability

The data presented in the study are deposited in the GEO repository, accession number GSE294491.
