# Correction: Renoprotective effects of GHRH agonist MR409 is associated with reduced oxidative stress and ferroptosis in diabetic mice

**DOI:** 10.3389/fphar.2025.1737333

**Published:** 2025-11-12

**Authors:** Yueyang Liu, Rong Fu, Qi Tang, Yaoxia Zhang, Ruiping Cai, Limin Liu, Hui Jia, Junjia Gao, Ming-Sheng Zhou

**Affiliations:** 1 Shenyang Key Laboratory of Vascular Biology, Science and Experimental Research Center of Shenyang Medical College, Shenyang, China; 2 Department of Physiology, Shenyang Medical College, Shenyang, China; 3 Department of Cardiology, 2nd Affiliated Hospital Shenyang Medical College, Shenyang, China; 4 School of Traditional Chinese Medicine, Shenyang Medical College, Shenyang, China

**Keywords:** growth hormone-release hormone analogue, diabetic nephropathy, oxidative stress, ferroptosis, Klotho

There was a mistake in Figure 3G as published. The images for the MR409-treated group and the control group were inadvertently duplicated due to a file handling mistake during figure preparation. This was an unintentional error and does not reflect the actual experimental results, which were correctly recorded and remain valid. The corrected Figure 3 appears below.

**FIGURE 3:**
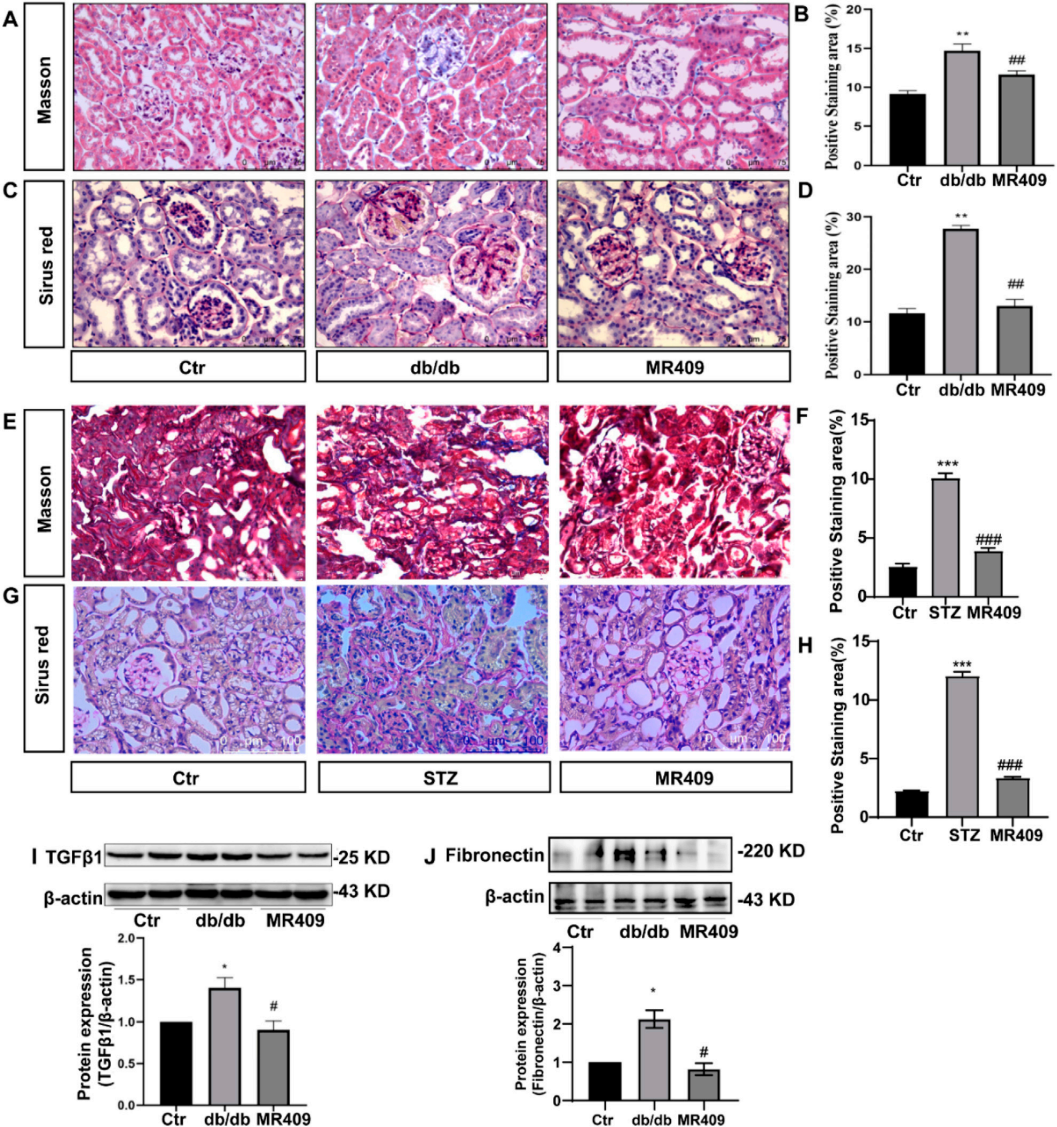
MR409 attenuates the renal fibrosis in db/db and STZ-induced diabetic mice. Representative images of renal sections from db/db mice by Masson staining **(A)** and Sirus red staining **(C)**, and correspondence semiquantitative analysis of positive staining area **(B,D)**. Representative images of renal sections from STZ mice by Masson staining and Sirus red staining **(E,G)**, and correspondence semiquantitative analysis of positive staining area **(F,H)**. Protein expressions of TGFβ1 and Fibronectin in the kidney of db/db mice **(I,J)**. Data are expression as mean ± SEM. ***P < 0.001, **P < 0.01, *P < 0.05 vs. control (Ctr) group; ###P < 0.001, ##P < 0.01, #P < 0.05 vs. db/db group or STZ group. n = 6. Scale bar = 75 μm in panels **(A,C,G)**, Scale bar = 100 μm in panels **(E)**.

The original article has been updated.

